# Comparison of HIV Testing among Children and Adults with Tuberculosis, Vietnam

**DOI:** 10.4236/jtr.2017.54030

**Published:** 2017-12-29

**Authors:** Tyson Volkmann, Binh Nguyen, Ebelechukwu G. Anyalechi, Kimberly N. Chapman Hedges, Ho Van Anh, Pham Quang Tue, Nguyen Viet Nhung, Eleanor S. Click

**Affiliations:** 1 Division of Global HIV and TB, Centers for Disease Control and Prevention, Atlanta, USA; 2 Division of Tuberculosis Elimination, Centers for Disease Control and Prevention, Atlanta, USA; 3 Vietnam National Tuberculosis Program, Hanoi, Vietnam

**Keywords:** Surveillance, Operational Research, Childhood, TB, Pediatric

## Abstract

HIV testing among persons with tuberculosis (TB) results in high-yield identification of persons infected with HIV. To evaluate differences in HIV testing among children versus adults with TB in Vietnam, we collected and analyzed age-disaggregated facility and aggregated provincial data from the National Tuberculosis Program. HIV testing was incompletely documented for >70% of children, whereas adult testing data were >90% complete. Standardized training of personnel for universal HIV testing and documentation for children with TB could improve HIV case-detection and permit linking of children with HIV to antiretroviral treatment to prevent morbidity and mortality.

## 1. Introduction

Children account for up to 40% of tuberculosis (TB) cases in high-burden settings, and those diagnosed with TB are more likely to be HIV-infected than children without TB [1]. Historically, children with HIV are less likely to receive antiretroviral therapy (ART) relative to adults [2], with current estimates of <50% globally [3]. Routine HIV testing of children with TB could facilitate HIV case finding and linkage to HIV care and treatment.

Given the high prevalence of HIV among people with TB in many countries, in 2007 the World Health Organization (WHO) recommended HIV testing for all persons with TB, including those who are newly-diagnosed and those that have already started TB treatment [4]. In 2013, WHO recommended starting antiretroviral therapy (ART) regardless of CD4 count for all patients with both TB disease and HIV infection [5]. Timely HIV diagnosis, care, and treatment among children with TB require integrated or collaborative TB and HIV services. TB surveillance systems should provide robust HIV testing data to detect and report cases of HIV among all patients with TB. Unfortunately, many HIV and TB surveillance systems do not report age-disaggregated data [6], limiting both knowledge of the epidemiology of pediatric TB/HIV and assessment of the extent to which recommendations for HIV testing of children with TB are implemented.

As part of an evaluation of the national childhood TB/HIV surveillance system in Vietnam, we collected HIV testing data for children and adults prescribed TB treatment within the National Tuberculosis Program (NTP) in five highly-populated provinces in Vietnam. In this short report, we present results of a comparison of completeness of these data among children and adults.

## 2. Methods

Five geographically diverse provinces in Vietnam with relatively high TB burden—Can Tho, Hai Phong, Ha Noi, Ho Chi Minh City, and Khanh Hoa—were selected for this evaluation. With a combined population of nearly 20 million people, they account for23.2% of the national population.

In 2014, we collected two sources of data on completeness of HIV testing among persons registered for TB treatment. HIV testing was conducted according to the national algorithm, and results were documented in the TB register. First, we collected patient-level data for all children < 15 years old treated for TB during 2011–2012 from the paper TB treatment registers in every public inpatient or outpatient NTP facility within the selected districts. Data were abstracted from TB registers and entered into a single database. We then de-duplicated the data by matching multiple records for the same individual based on a child’s name, address, age, sex, registration date, date of birth, and demographic data. No patient -level data were collected for adults.

Second, we collected aggregated data from the NTP annual report for each of the five provinces for 2011 and 2012 for all-aged patients (including adults), representing all the data from the combined facility registers. NTP-aggregated data are reported by age group, sex, TB type, and HIV status. However, for patients with smear negative TB, age-stratified data are available for both HIV testing and HIV status, but HIV status is only reported for patients with smear-positive TB. Stratified aggregated data from all five provinces were combined.

To determine the completeness of HIV testing and HIV reporting among children with smear-negative and smear-positive TB, we analyzed individual-level facility data. We then compared HIV testing information aggregated at the provincial level among children and adults with smear-negative TB.

The CDC’s National Center for HIV/AIDS, Viral Hepatitis, STD, and TB Prevention determined that this was a routine program evaluation that was not research and did not require review by a human subjects institutional review board. The protocol was ethically approved by Vietnam’s National Lung Hospital Scientific Board.

## 3. Results

After de-duplication of data, 721 cases of TB were reported for children during 2011–2012 from 76 facility-level registers across five provinces. Of these, 9 (1%) were documented to have known HIV positive status at time of TB clinic registration and 197 (27%) children had documentation of results of HIV testing performed at the TB clinic; of these 197 children tested for HIV, 5 (3%) tested newly positive (Table 1). HIV testing data were highly variable across province and facility. Overall, a high percentage of the total sample of children with TB had missing data for TB diagnostic tests (e.g., smear [40%], culture [96%], radiography [70%]). Facility data completeness for TB diagnostic tests varied from 0 to 100% for smear, culture, and radiography within each province.

Provincial-level aggregated data (Table 2) demonstrate a discrepancy between the proportion of children versus adults registered for TB treatment who were tested for HIV. Among NTP aggregate reports for patients with TB diagnosed with smear-negative pulmonary TB registered for treatment in 2011–2012, an HIV test result was documented for 6.8% of children under five years old, 20.1% of children aged five to 14, and 92.2% of adults ≥15. Differences in percentages between adults and children were statistically significant at p < 0.001. The percentages of those tested for HIV did not vary significantly by TB disease location (*i.e*., pulmonary versus extrapulmonary).

## 4. Discussion

Based on our evaluation, there were many missed opportunities to identify new HIV cases among children registered for TB treatment within the NTP and to link these children with TB/HIV to life saving antiretroviral treatment. If we assumed all children had the same risk of HIV and applied the 2.5% positivity among the 515 children with unknown HIV status, an additional 13 cases of newly-diagnosed HIV might have been identified.

Although the age-disaggregated HIV testing data for comparison of children versus adults were available for smear-negative patients only, we noted a pattern of incomplete HIV test results for children that was consistent with what we found for all children with TB using patient-level data. We were unable to determine whether the primary reason for the low proportion of children with a documented HIV test result is due to lack of testing or lack of documentation, but high levels of documented test results among adults suggests the primary gap is in testing children for HIV. Further work to understand why documented testing of HIV among children is lower than adults should be undertaken to eliminate this gap and ensure all children with TB have a documented HIV status.

Documentation of HIV testing of all children with TB should be supported by the national TB program. Current WHO reporting frameworks do not provide specific tools for documentation of HIV testing among people with TB [7]. Testing documentation could include the type of test received (e.g., rapid, PCR) to ensure all children receive the appropriate age-specific test. During our assessment, the facility registers did not provide information about which type of test was conducted, so it is unknown whether a definitive test (PCR) was performed for children <18 months. Completeness of HIV test result did not significantly vary for children when stratified at the age of 18 months, but without more detailed test information, we were determine whether appropriate testing took place. Given the WHO recommendation to treat all persons living with HIV with ART [8], it is important that all children living with HIV are identified, know their status, and are started on ART. Recent efforts to increase childhood ART coverage through targeted funding for greater access to HIV medicines for children and development of pediatric ART formulations have been undertaken [9]. It is important to test all children with TB for HIV so that children testing positive can receive ART.

## 5. Conclusion

Although the percentage of adults with TB tested for HIV in the five selected provinces in Vietnam was >90% (but short of the 100% recommended by WHO), HIV testing for children with TB in 2011 and 2012 was not documented for the majority of children. All children with TB disease should be tested for HIV, their results should be recorded and reported via surveillance, and children testing positive should be referred for HIV care and treatment. A coordinated TB/HIV programmatic approach should be enhanced, and the systems for HIV testing that are currently in place for adults that facilitate >90% HIV testing in those with TB should extend to children to ensure universal testing, care, and treatment across Vietnam. This could include training for TB personnel in health facilities to follow national childhood HIV testing guidelines in order to ensure universal HIV testing and 100% linkage to HIV treatment among children.

## Figures and Tables

**Table 1:** Number of TB cases and HIV testing among children < 15 diagnosed with TB, facility registers, NTP, five provinces, Vietnam, 2011 and 2012^1^.

	Total, 2011–2012 n (%)
**All TB cases (from 76 facilities)**	721
**Cases by age group**	
0 – 4	331 (45.9)
5 – 14	390 (54.1)
**HIV counseling and testing**	
Documentation that neither counselling nor test received	30 (4.2)
Counselling documented but HIV test result not documented	20 (2.7)
Known HIV positive upon entry to TB care	9 (1.2)
**HIV test result documented**	
HIV negative	192 (26.6)
Newly HIV positive	5 (0.7)
No documentation of HIV counseling or testing	465 (64.5)

1 Duplicate cases removed according to duplication elimination protocol.

**Table 2:** Total number of acid-fast bacilli smear-negative^1^ tuberculosis cases reported by type and age, and number of persons with TB who underwent HIV testing and were HIV-positive, aggregated quarterly reports, five provinces, Vietnam, 2011 and 2012^2^.

	Pulmonary			Extrapulmonary		

5-Province Total	Age					

	0 – 4	5 – 14	≥15	0 – 4	5 – 14	≥15
Number of patients HIV tested/total cases (percent tested of total cases)	16/235 (6.8)	28/139 (20.1)	7948/8623 (92.2)	39/155 (25.2)	67/237 (28.3)	7623/8308 (91.8)
Number HIV positive of those tested (percent positive of those tested)	2 (12.5)	0 (0.0)	693 (8.7)	1 (2.6)	1 (1.5)	955 (12.5)

1 Age-disaggregated data for HIV status for children with smear-positive TB was included in the NTP reporting form, but HIV testing result was not available for children with smear-positive TB.

2 All differences between the ≥ 15 age group and the younger age groups are statistically significantly at p < 0.001.
